# Nitridochromate(IV): LiSr_2_[CrN_3_]

**DOI:** 10.1021/acs.inorgchem.3c01697

**Published:** 2023-08-03

**Authors:** Natalia Gloriozova, Yurii Prots, Franziska Jach, Mitja Krnel, Matej Bobnar, Alim Ormeci, Yuri Grin, Peter Höhn

**Affiliations:** † Max-Planck-Institut für Chemische Physik fester Stoffe, Nöthnitzer Straße 40, 01187 Dresden, Germany; ‡ Faculty of Chemistry and Food Chemistry, Technische Universität Dresden, 01062 Dresden, Germany

## Abstract

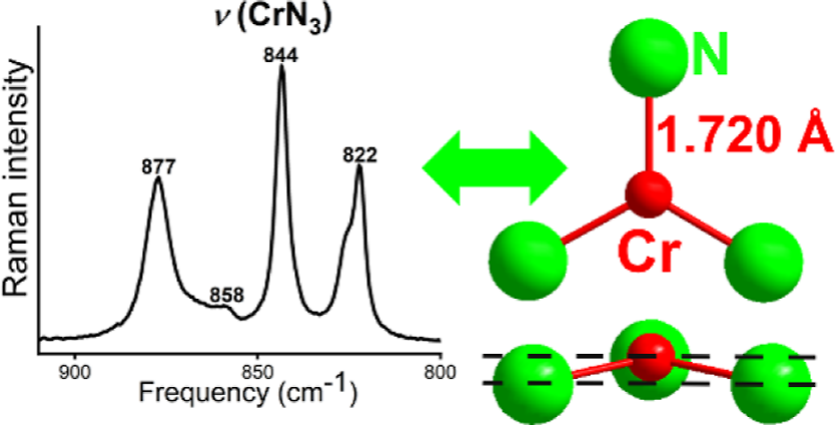

The quaternary nitridochromate(IV) LiSr_2_[CrN_3_] crystallizes in a new structure type with the non-centrosymmetric space group *P*2_1_ (no. 4) with *a* = 5.5685(7) Å, *b* = 5.3828(8) Å, *c* = 7.5381(1) Å, and β = 92.291(8)°. Predominant structural features of the compound are slightly nonplanar trigonal units [CrN_3_]^5–^, which are connected by three-fold coordinated lithium to form slabs in the (001) plane. Shorter Cr–N bond lengths in comparison with reported nitridochromates(III), as well as diamagnetic behavior and vibrational spectroscopy data indicate Cr(IV), which is in a good agreement with the charge balance. According to electronic structure calculations, the compound is a semiconductor with a band gap of 1.19 eV.

## Abstract 2

The di-cationic nitridometalate LiSr_2_[CrN_3_] is an example of almost elusive nitridochromates(IV) with slightly pyramidal trigonal units [CrN_3_]^5−^. Magnetic measurements, vibrational spectroscopy, as well as shorter Cr−N bond lengths in comparison with reported nitridochromates indicate the presence of Cr(IV). LiSr_2_[CrN_3_] is nonmagnetic and a semiconductor according to band structure calculations. The bonding situation is characterized by highly polar N-dominated N−Cr and Cr-dominated multi-atom bonds.

## Introduction

Ternary and multinary nitridochromates of alkali and alkaline-earth elements exhibit oxidation states between III and VI for chromium, whereas in binary chromium nitrides, only a maximum of Cr(III) at ambient pressures is observed.^1,2^ Depending on the nitrogen partial pressure in the system, the highest oxidation states (V, VI) can be reached in a combination with lithium and alkaline-earth metals. These compounds typically consist of isolated tetrahedral anions [CrN_4_]^*x*−^ or edge-sharing tetrahedra with chromium–chromium bonding. Up to now, the following Cr(V) and Cr(VI) nitridometalates are known: Li_4_Sr_2_[Cr_2_^V^N_6_],^3^ Ca_4_[Cr_2_^V^N_6_], Sr_4_[Cr_2_^V^N_6_],^4^ Ba_5_[Cr^V^N_4_]N,^5^ Li_6_[Cr^VI^N_4_], Li_15_[Cr^VI^N_4_]_2_N,^6^ and Sr_3_[Cr^VI^N_4_].^7^ Additionally, only a few compounds with Cr(III) in (distorted) trigonal planar coordination and an alkaline-earth metal as a cation are reported: Ca_3_[Cr^III^N_3_],^8^ Sr_3_[Cr^III^N_3_], and Ba_3_[Cr^III^N_3_].^9^ Recent studies suggest that some of these compounds may be described by balances with excess electrons Sr_3_[Cr^IV^N_3_]·e^–^ and Ba_3_[Cr^IV^N_3_]·e^–^,^10^ which are able to attach hydrogen, forming multianionic nitridochromate-hydrides Sr_3_[Cr^IV^N_3_]H and Ba_3_[Cr^IV^N_3_]H,^11^ respectively. Moreover, the nitridochromate–hydride Ca_6_[Cr_2_^III,IV^N_6_]H,^12^ which contains an ethane-like anion, provides another example for mixed-valent/intermediate valent Cr(III)/Cr(IV) species and a direct Cr–Cr interaction. However, to our knowledge, no “pure” nitridochromates(IV) are described until now. Here, we report on the synthesis and characterization of a new multi-cationic nitridochromate LiSr_2_[CrN_3_], which contains Cr(IV), and trigonal planar units [LiN_3_], forming slabs in the (001) plane.

## Experimental Section

Single crystals of LiSr_2_[CrN_3_], showing dark gray metallic lustre, were synthesized applying a modified high-temperature centrifugation-aided filtration technique (HTCAF).^13,14^ A mixture of Sr_2_N, Cr, Li_3_N, and Li in molar ratio 1:1.8:2.8:17 was sealed in a tantalum ampule with integrated sieve and subsequently heated at 1023 K for 2 h. After cooling down to 573 K, the mixture was centrifuged to allow for proper separation of crystals from the flux.

To check a possible phase width and potential other phases in the system, several more experiments were performed using the same HTCAF technique, but employing different starting materials, molar ratios, or thermal regimes (Table S1). In all of the experiments, crystals of LiSr_2_[CrN_3_] were obtained, among varying amounts of Sr_3_[CrN_3_], Sr_3_[CrN_3_]H, and other minor phases.

Nearly single-phase samples of dark gray microcrystalline LiSr_2_[CrN_3_] were synthesized from pelletized mixtures of Sr_2_N, CrN, and Li_3_N with molar ratio 0.7:1:2.6 in sealed tantalum ampules at 1023 K for 36 h. Several subsequent cycles involving grinding, adding excess of Sr_2_N and Li_3_N, and re-heating at the same temperature for longer times led to nearly single-phase samples of LiSr_2_[CrN_3_] with a small amount of unspecified impurities. High temperatures and long annealing time may lead to evaporation of light elements and decomposition of alkaline-earth nitrides, as well as reactions with the crucible material; therefore, the reaction tubes were arc-sealed. Powder X-ray diffraction was used to determine sample purity. The Rietveld refinement was conducted using the Jana2006^15^ software on the powder pattern of the samples (Figure S1).

Detailed information on synthesis, crystal structure determination, and physical properties determination can be found in the Supporting Information (see Experimental details). Details of the data collection and further crystallographic information are listed in Table 1. Further details of the crystal structure investigations may be obtained from FIZ Karlsruhe, 76,344 Eggenstein-Leopoldshafen, Germany (fax (+49)7247-808-666; e-mail: crysdata@fiz-karlsruhe.de, on quoting the deposition numbers CSD-2264610).

**Table 1:** Crystallographic Information for LiSr_2_[CrN_3_]

crystal system	Monoclinic
space group	*P*2_1_ (#4)
*a* (Å)	5.5685(7)
*b* (Å)	5.3828(8)
*c* (Å)	7.5381(1)
β (deg)	92.291(8)
*V* (Å^3^)	225.77(5)
*Z*	2
ρ_calc_ (g/cm^3^)	3.64
μ (mm^–1^)	16.32
2θ range (deg)	8.932–67.09
diffractometer	RIGAKU AFC7
wavelength (Å)	0.71069 (Mo *K*α)
monochromator	graphite
temperature (K)	293(2)
measured reflections	1879
unique reflections	1299
*R*_int_	0.0229
observed reflections [*F*_o_ > 4σ(*F*_o_)]	1205
refined parameters	65
extinction	0.008(5)
*R*_1_ [*F*_o_ > 4σ(*F*_o_)]	0.0394
*R*_1_ (all data)	0.0415
w*R*_2_	0.0982
goodness-of-fit on *F*^2^	1.052
Flack parameter	–0.02(2)
residual peaks (e Å^–3^)	–1.93/1.78

## Results and Discussion

### Crystal Structure

**Figure 1:**
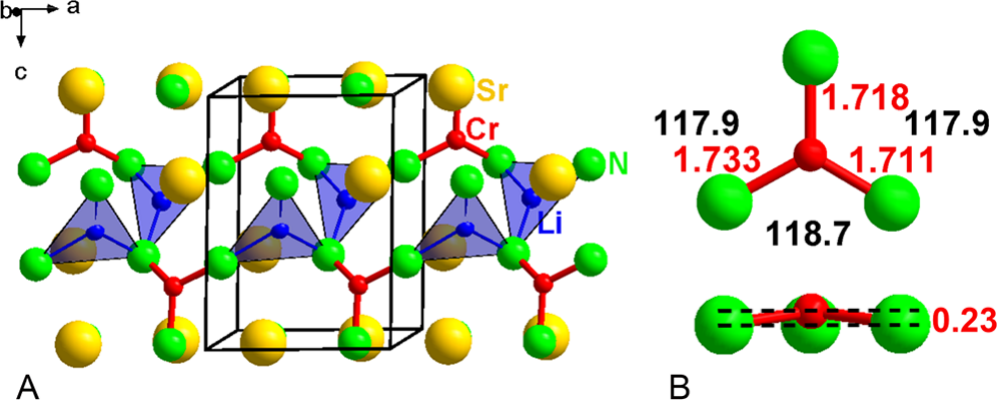
(A) Crystal structure of LiSr_2_[CrN_3_]. (B) [CrN_3_]^5–^ complex anion with bond lengths depicted in red (Å) and N–Cr–N bond angles depicted in black (°).

The title compound crystallizes in a monoclinic lattice, space group *P*2_1_ (no. 4) with *a* = 5.5685(7) Å, *b* = 5.3828(8) Å, *c* = 7.5381(1) Å, and β = 92.291(8)° with 1 Cr, 1 Li, 2 Sr, and 3 N symmetry-independent positions. The refined atomic coordinates and equivalent displacement parameters, as well as the anisotropic displacement parameters, are listed in Tables S2 and S3. The predominant structural feature in LiSr_2_[CrN_3_] is the slightly pyramidal trigonal [CrN_3_]^5–^ group with pseudo *C*_3*v*_ symmetry. Chromium atom is located about 0.23 Å out of the plane defined by the three coordinating nitrogen atoms (Figures 1, S2). A similar pyramidal unit [*M*N_3_]^6–^ is observed in Li_24_[Mn^III^N_3_]_3_N_2_,^16^ whereas Ba_3_[Cr^III^N_3_], Sr_3_[Cr^III^N_3_],^9^ Sr_3_[Cr^IV^N_3_]H,^10^ and Ba_3_[Cr^IV^N_3_]H^11^ contain trigonal planar [CrN_3_]^6–^ and [CrN_3_]^5–^ units of *D*_3*h*_ symmetry, and in Ca_3_[Cr^III^N_3_],^8^ the planar [CrN_3_]^6–^ units feature *C*_2*v*_ symmetry, respectively.

The average Cr–N distance in LiSr_2_[CrN_3_] is 1.719(4) Å, which is slightly shorter than in reported nitridochromates(III), supporting the presence of Cr(IV) in the structure (Table 2).

**Table 2:** Selected Interatomic Distances for [CrN_3_]^*x*−^ and [LiN_3_] Fragments in LiSr_2_[CrN_3_] and Related Compounds

fragment	compound	average *d*(Cr–N)/Å	fragment	compound	average *d*(Li–N)/Å
[CrN_3_]^*x*^^–^	LiSr_2_[Cr^IV^N_3_]	1.719(4)	[LiN_3_]	LiSr_2_[Cr^IV^N_3_]	2.03(1)
	Ca_3_[Cr^III^N_3_]^8^	1.766(7)		Li3N^17^	2.1060(4)
	Sr_3_[Cr^III^N_3_]^9^	1.728(3)		LiCaN^18^	2.0955(5)
	Sr_3_[Cr^IV^N_3_]H^10^	1.709(7)		Li4SrN218	2.136(7)
	Ba_3_[Cr^III^N_3_]^9^	1.732(8)		Li_24_[Mn^III^N_3_]_3_N_2_^16^	2.105(3)
	Ba_3_[Cr^IV^N_3_]H^11^	1.739(3)			

**Figure 2:**
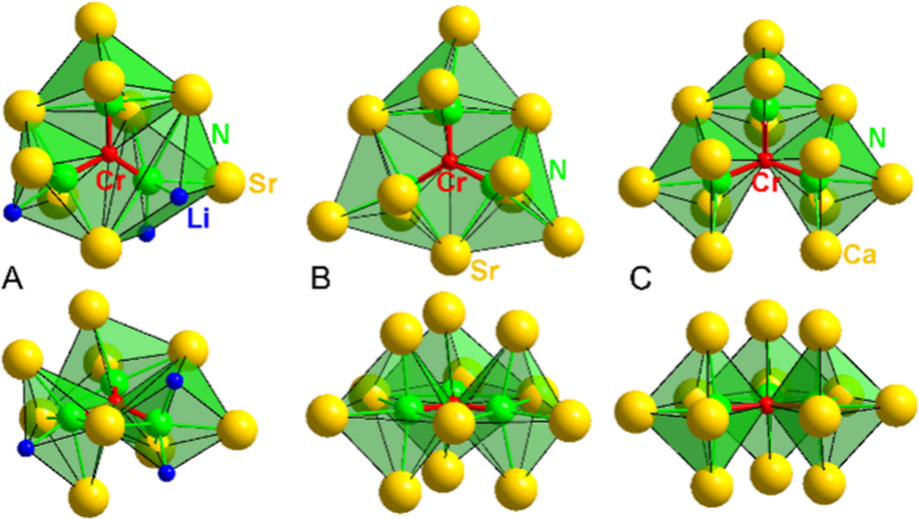
Coordination of [CrN_3_]^*x*−^ units in (A) LiSr_2_[CrN_3_], (B) Sr_3_[Cr^III^N_3_], and (C) Ca_3_[Cr^III^N_3_].

There are three different types of environments of the N species in the [CrN_3_]^5–^ anions in LiSr_2_[CrN_3_]: NSr_2/2_Sr_2_LiCr_1/3_, NSr_3/2_Sr_2_Cr_1/3_, and NLi_2_Sr_3/2_SrCr_1/3_, sharing common edges (Figure 2A). In Sr_3_[Cr^III^N_3_], the environment of nitridochromate anion is formed by three edge-sharing NSr_2/2_Sr_3_Cr_1/3_ octahedra.^9^ However, the coordination in Ca_3_[Cr^III^N_3_] is completely different and consists of one NCa_2/2_Ca_3_Cr_1/3_ and two NCa_1/2_Ca_4_Cr_1/3_ polyhedra, sharing edges and leaving an “open face”.^8^

In LiSr_2_[CrN_3_], lithium atoms are surrounded by three nitrogen atoms, forming slightly distorted trigonal [LiN_3_] units. Lithium atom is located about 0.11 Å out of the plane defined by the three coordinating nitrogen atoms. Similar trigonal arrangements are observed in Li_3_N,^17^ LiCaN, and Li_4_SrN_2_,^18^ whereas in the quaternary nitridochromate, Li_4_Sr_2_[Cr_2_^V^N_6_]^3^ tetrahedral [LiN_4_] units are present. In Li_24_[Mn^III^N_3_]_3_N_2_,^16^ both trigonally and tetrahedrally coordinated lithium atoms are observed. In the title compound, [LiN_3_] units are linked by shared nitrogen atoms, forming zig-zag chains along [010] direction, which are connected by [CrN_3_]^5–^ groups along [100], thereby forming slabs in the (001) plane.

The average Li–N distance is 2.03 Å, which is slightly shorter than in other trigonal planar coordinated lithium units in Li_3_N,^17,19^ LiCaN,^18^ Li_4_SrN_2_,^18^ and Li_24_[Mn^III^N_3_]_3_N_2_^16^ (Table 2). Strontium atoms are six- and seven-fold coordinated by nitrogen (Figure S3). Sr–N distances are similar to those of 2.67–2.85 Å in Sr_3_[Cr^III^N_3_]^9^ and 2.69–3.18 Å in Sr_4_[Cr_2_^V^N_6_]^4^ (Table S4). N1 and N2 are octahedrally coordinated (Figure S3), which is also observed in Ca_3_[Cr^III^N_3_],^8^ Ca_6_[Cr_2_^III,IV^N_6_]H,^12^ Sr_3_[Cr^III^N_3_], Ba_3_[Cr^III^N_3_],^9^ Ca_4_[Cr_2_^V^N_6_], Sr_4_[Cr_2_^V^N_6_],^4^ and Ba_5_[Cr^V^N_4_]N.^5^ In contrast, N3 is seven-fold coordinated, with shorter N–Li and N–Cr contacts. Similar arrangements are found in LiMgN,^20^ LiCaN, Li_4_SrN_2_,^18^ and Li_3_BN_2_.^21^ Cr–Cr distances range from 4.985(2) to 5.678(2) Å and are much too long for M–M bonding or significant direct exchange interaction, but are similar to average Cr–Cr distances in Ca_3_[Cr^III^N_3_] (5.03 Å),^8^ Sr_3_[Cr^III^N_3_] (5.250 Å), or Ba_3_[Cr^III^N_3_] (5.475 Å).^9^ In these compounds, Cr–Cr distances increase from calcium to barium according to increasing cation size.

**Figure 3:**
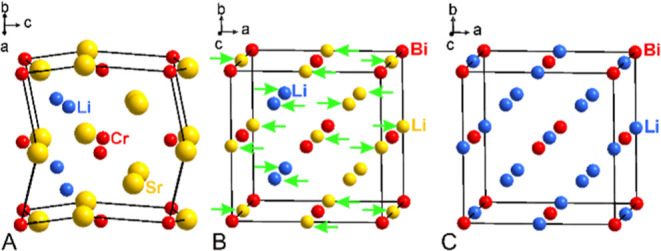
Crystal structure of LiSr_2_[CrN_3_], considering only metal positions (A), and its relation to the Li_3_Bi-type (C) by shifting the atomic positions (B).

Considering the metal positions only, the crystal structure of LiSr_2_[CrN_3_] is related to the Li_3_Bi-type arrangement with the [CrN_3_]^5–^ units forming a cubic closest packing pattern. All octahedral voids as well as half of the tetrahedral voids are occupied by Sr2, and Sr1, respectively, the remaining tetrahedral voids are filled with lithium. The whole setup is somewhat distorted (Figure 3).

A similar arrangement is observed in CaBa_2_[WN_4_],^22^ where tetrahedral units [WN_4_]^6–^ are arranged in a cubic closest pattern, and all octahedral voids as well as half of tetrahedral voids are occupied by barium and the remaining tetrahedral voids by calcium. However, the arrangements of the atoms in the tetrahedral voids are different than in LiSr_2_[CrN_3_].

### Electronic Structure and Chemical Bonding

**Figure 4:**
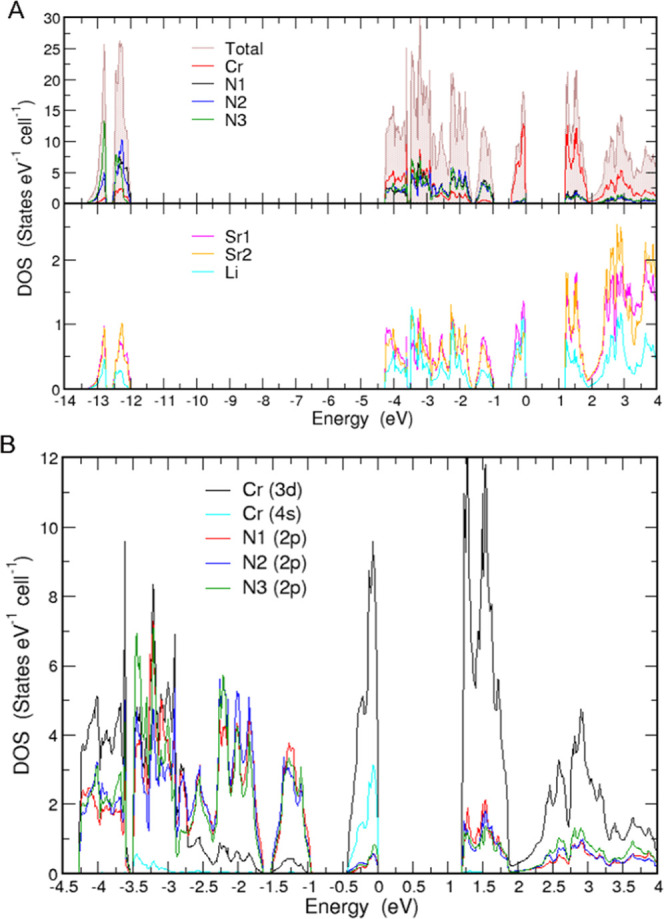
(A) Computed total and atom-resolved electronic density of states (DOS) for LiSr_2_[CrN_3_]. The upper panel presents the total DOS as well as Cr and N atom contributions to it. The lower panel presents Sr and Li contributions. (B) Projected DOS showing Cr 3d, 4s, and N 2p contributions in the energy range [−4.5, 4.0] eV.

The spin-polarized calculations for LiSr_2_[CrN_3_] converged to the zero-moment solution implying the absence of a long-range magnetic order. Hence, all the results presented here are based on non- spin-polarized calculations. LiSr_2_[CrN_3_] is a semiconductor with a band gap of 1.19 eV according to the computed electronic density of states (DOS) (Figure 4A). The states between −13.35 and −12.0 eV are mainly due to the N 2s electrons; there are two electrons in the lower part (between −13.35 and −12.75 eV) and four in the upper (between −12.55 and −12.0 eV) per formula unit. The former is dominated by N3 (0.92 electrons) and the latter by N1 and N2 (1.32 electrons). The remaining 20 valence electrons occupy the states between −4.5, and 0.0 eV. Cr 3d, 4s, and N 2p make the most relevant contributions in this range (Figure 4B). Cr 3d contributions dominate from −4.3 to −2.75 eV. The DOS between −2.75 and −1 eV is dominated by N 2p. The topmost valence bands are very narrow with a width of 0.43 eV and formed mainly by Cr 3d and 4s orbitals (more on this later).

**Figure 5:**
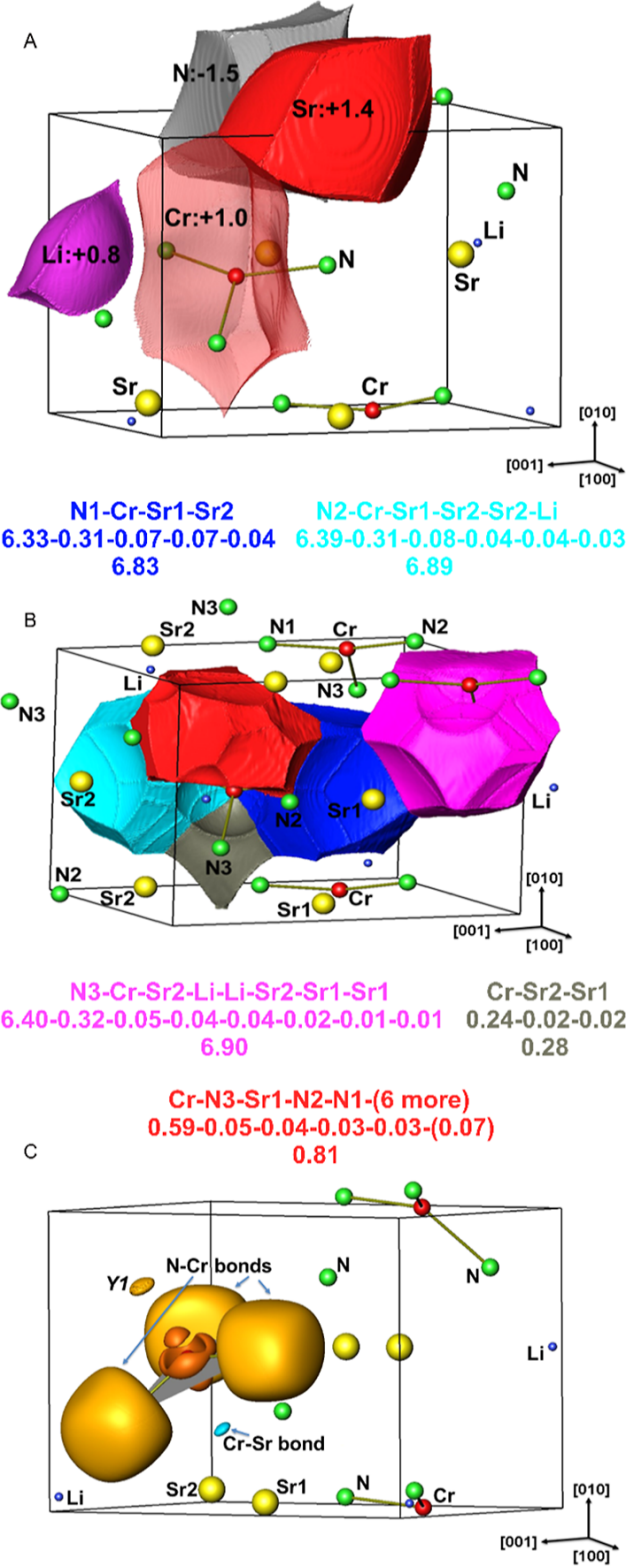
Chemical bonding in LiSr_2_[CrN_3_]: (A) QTAIM shapes of atomic basins and the effective charges. (B) ELI-D bond basins with information on participating atoms (first line), their individual contributions (second line), and the total basin populations (third line for each atom). A spherical surface is typically a common boundary with a core basin; the flat surfaces are boundaries with other bond basins. (C) ELI-D distribution showing the bonding situation in the [CrN_3_]^5–^ anion. The isosurface values are 1.425 (dark orange), 1.305 (orange), and 1.050 (cyan) depicting the Cr core, N–Cr bonds and Y1, and Cr–Sr1–Sr2 bond, respectively. Y1 denotes the Cr-dominated 11-atomic bond.

The ionic part of the atomic interactions in the title compound was studied by computing the charge transfer. The application of the QTAIM method^23^ yields the following effective charges: both Sr atoms are 1.4+, Cr is 1.0+, Li is 0.8+, N1 is 1.5–, and N2 and N3 are 1.6–. Additionally, the shapes of the QTAIM basins provide qualitative information on the nature of bonding for each atom (Figure 5A). If the electrons of the last shell are completely transferred to the other atoms, the QTAIM shape involves the inner shells only, and the QTAIM basin assumes a round, spherical shape. The QTAIM basin of an atom participating in mainly two-atomic covalent bonds presents planar or almost planar faces that are perpendicular to the interatomic line between the bonded atoms. The N and Cr QTAIM basins shown in Figure 5A are good examples for the latter case. The Cr QTAIM basin was plotted in transparent mode so that Cr–N interatomic lines are visible. It is noteworthy to observe that the concave surfaces of the Cr QTAIM basin facing the two Sr atoms suggest polar covalent Cr–Sr interactions. In line with the above explanation, the atom with the concave surface provides more electrons to the bond than the neighboring atom having the convex round surface.

A deeper understanding of the nature of atomic interactions can be achieved by the topological analysis of the electron-localizability indicator in the ELI-D representation. The maxima (attractors) of the ELI-D in the valence region and the associated basins are used to identify the bonding interactions. The atomic interactions in LiSr_2_[CrN_3_] can be divided into two groups (Figure 5B,C). The first group belongs to the Cr–N interactions. Intuitively, one expects separate ELI-D maxima for the Cr–N bonds and for the lone pairs at nitrogen atoms. Due to the small contributions of the chromium in comparison with nitrogen, the formation of separate attractors on the Cr–N contacts is suppressed, and only one bond basin appears in the vicinity of each nitrogen atom (pink, light-blue and dark-blue in Figure 5B for N3, N2, and N1, respectively). Such merging behavior of bond attractors toward “lone-pair” attractors was already found not only in nitridometalates Li_6_Ca_2_[Mn_2_N_6_],^24^ Li_6_Sr_2_[Mn_2_N_6_],^25^ or ammonia molecule NH_3_,^26^ but also in other substances with high electronegativity difference like Mg_3_Pt_2_,^27^ Eu_3_Ga_2_,^28^ and K_3_Bi_2_.^29^ The basin populations are 6.83, 6.89, and 6.90 electrons for N1–Cr, N2–Cr, and N3–Cr basins, respectively. The bond polarities, estimated according to the position space approach,^30^ are 0.936, 0.927, and 0.928 on the scale between 0 (covalent) and 1 (ionic) for N1–Cr, N2–Cr, and N3–Cr, respectively. N atoms provide about 92.7% and Cr 4.5% of these basin populations, with the remaining electrons being contributed by 3, 4, or 6 atoms, respectively. Note that the total number of electrons each N type has in the valence region is 6.36, 6.43, and 6.46 for N1, N2, and N3, respectively. The second group consists of two Cr-dominated interactions. One of these contains 0.81 electrons of which 0.59 (72.6%), 0.05 (5.4%), and 0.04 (4.2%) are provided by Cr, N3, and Sr1, respectively. The remaining contributions by 8 atoms (3 × N, 3 × Sr, and 2 × Li) add up to 0.13 electrons. We label this basin as Cr-dominated 11-atomic interaction. The other one is a 3-atomic Cr–Sr2–Sr1 interaction (as hinted by the shape of the Cr atomic basin) with individual contributions of 0.24 (Cr), 0.02 (Sr2), and 0.02 (Sr1) electrons adding up to the total bond population of 0.28. Both of these interactions can also be regarded as lone-pair-like features due to the large Cr contributions. The attractors of these Cr-dominated interactions are located above and below the N1–N2–N3 plane, and the line connecting the attractors is perpendicular to this plane. Therefore, it is illuminating to analyze the electronic structure with the quantization axis (the *z*-axis for the orbital angular momentum operator) taken perpendicular to one of the two N1–N2–N3 planes in the unit cell. The remarkable result is that the band giving rise to the DOS between −0.43 and 0 eV (Fermi level set to 0 eV) is made up of mainly the Cr 4s and 3d (3*z*^2^–*r*^2^) orbitals (Figure S4). Identical results were reported earlier for Ba_3_[Cr^IV^N_3_]H.^11^ Using the fact that this band is well isolated from the rest, we computed the electron density due to only this band, ρ_top_. Integration of ρ_top_ in the whole unit cell gives two electrons per formula unit. It can also be integrated separately inside each ELI-D basin to get its contribution to the electron populations of the bonds and core shells. Of these two electrons, 0.42 and 0.10 are found in the basins of the Cr-dominated 11-atomic bond and Cr–Sr1–Sr2 bond, respectively, accounting for the 52 and 36% of the bond populations, respectively. Before we discuss the different roles of the 4s and 3d (3*z*^2^–*r*^2^) orbitals, we recall that in the ELI-D analysis the 3d electrons of Cr appear in the core region. The reason is that ELI-D yields the shell structure of atoms and the d electrons of transition metals belong to the penultimate shell. Hence, the Cr contributions to the two Cr-dominated bonds must come mostly from the 4s electrons. Now, noticing that the 3d (3*z*^2^–*r*^2^) orbital is essentially the only d orbital present in this energy range, a strong deviation of the ELI-D distribution in the penultimate shell (principal quantum number *n* = 3) from the spherical distribution is expected. Indeed, Figure 5C shows (i) two ELI-D localization domains along the local quantization axis on either side of the Cr atom, (ii) a three-lobed ELI-D localization domain around the Cr atom in the plane perpendicular to the quantization axis (dark-orange isosurfaces). The corresponding basins can be referred to as perpendicular outermost core basin and in-plane outermost core basin, respectively. Integrating ρ_top_ inside these we find 0.50 and 0.30 electrons, respectively. These must be mainly due to the 3d (3*z*^2^–*r*^2^) orbital.

In summary, the bonding in the anion [CrN_3_]^5–^ is characterized by strongly polar Cr–N interaction, leading even to the suppressing of the dedicated ELI-D attraction and formation of the common “lone-pair” + bond basin and lone-pair-like Cr-dominated multi-atom interactions.

### Vibrational Spectroscopy and Lattice Dynamics

**Figure 6:**
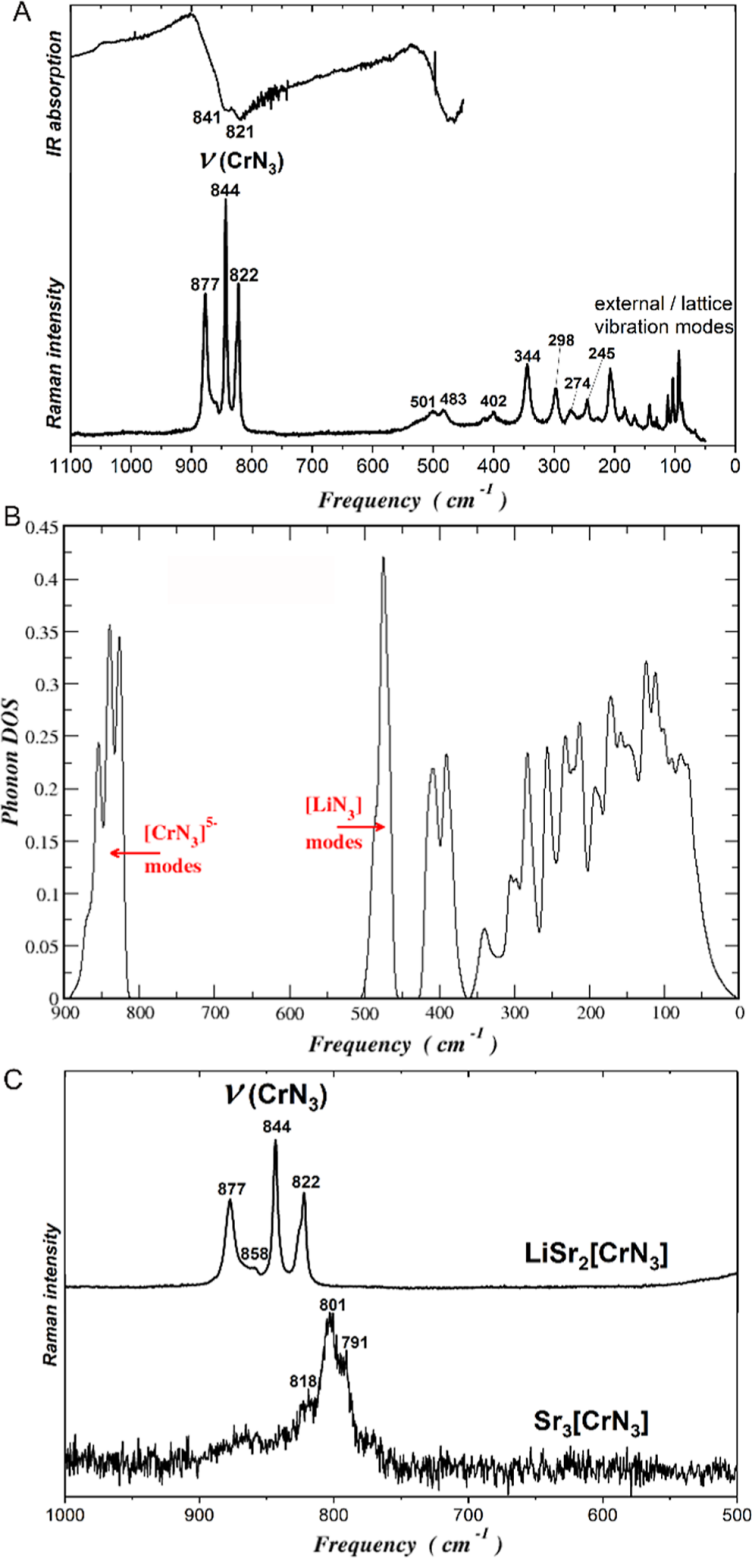
(A) IR (top) and Raman spectra (bottom) of LiSr_2_[CrN_3_]. (B) Computed phonon DOS for the fully-optimized LiSr_2_[CrN_3_]. The modes dominated by the vibrations of [CrN_3_]^5–^ and [LiN_3_] are identified. Maximum frequency is less than 900 cm^–1^. (C) Fragment of Raman spectra of LiSr_2_[CrN_3_] (top) and Sr_3_[CrN_3_] (bottom).

Infrared (IR) and Raman spectroscopy were used to investigate the nature of [CrN_3_]^5–^ anion in LiSr_2_[CrN_3_] (Figure 6A). According to point group *C*_3*v*_ of [CrN_3_]^5–^ anion, six υ(CrN_3_) modes in IR (3A, 3B), six υ(CrN_3_) modes in Raman (3A, 3B) spectra, six δ(NCrN) modes in IR (3A, 3B), and six δ(NCrN) modes in Raman (3A, 3B) spectra are expected by molecular site group analysis (for details, see Table S6).

The stretching modes of the anion [CrN_3_]^5–^ are located at 822–827 cm^–1^ and at 821–841 cm^–1^ in Raman and IR spectra, respectively. Only four and two bands out of the expected six are distinguishable. In Raman spectrum, two bands at 400 and 345 cm^–1^ are observed, which were assigned to deformation modes. Additionally, the bands above 3500 cm^–1^ are noticeable in all measured samples and reflect overtone or combination bands (Figure S5).

The phonon modes were calculated using the fully optimized unit cell. The optimized lattice parameters are *a* = 5.5712 Å, *b* = 5.4044 Å, and *c* = 7.5555 Å with β = 92.33°, yielding only 0.7% larger volume compared to the experimental value (Table 1). The calculated phonon DOS is shown in Figure 6B. The modes due to the anion [CrN_3_]^5–^ are the highest, lying between 810 and 900 cm^–1^. These stretching modes are followed by the modes of the [LiN_3_] units, located between 450 and 500 cm^–1^ and dominated by Li contributions (40–45% per Li atom). The modes involving the atoms of all three N types, Cr and Li, cover the range between 250 and 430 cm^–1^. Being the heaviest atom, Sr contributes mainly to modes below 200 cm^–1^.

The frequencies computed at the Γ point agree quite well with their experimental counterparts (Table S5).

Raman spectra were also measured for Sr_3_[Cr^III^N_3_] to be used as a reference (Figure 6C). In Sr_3_[Cr^III^N_3_], the ideal trigonal planar anion [CrN_3_]^6–^ has *D*_3*h*_ symmetry. Also, the phonon spectra of Sr_3_[CrN_3_] at the Γ point were calculated by a similar approach. In full agreement with the Raman measurements, the calculated [CrN_3_]^6–^ modes in Sr_3_[CrN_3_] shift to lower frequencies by 4–7%, 773–815 cm^–1^, in comparison to LiSr_2_[CrN_3_], reflecting the effect of the higher anion charge. Higher oxidation state Cr(IV) in LiSr_2_[CrN_3_] yields a stronger Cr–N interaction and therefore higher frequencies due to increased charge difference.

### Magnetic Properties

**Figure 7:**
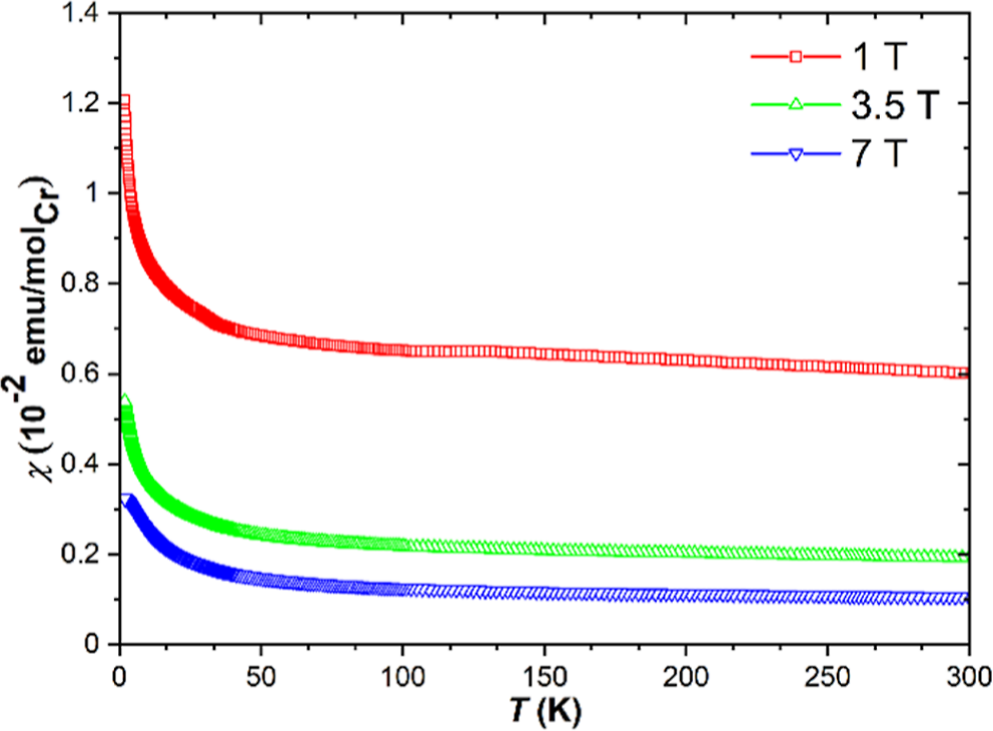
Temperature dependence of magnetic susceptibilities χ(*T*) of LiSr_2_[CrN_3_] measured in 1, 3.5, and 7 T magnetic fields for temperatures between 1.8 and 300 K.

The magnetization of the LiSr_2_[CrN_3_] polycrystalline sample was measured for temperatures between *T* = 1.8 and 300 K in μ_0_*H* = 1, 3.5, and 7 T magnetic fields. A predominantly ferromagnetic signal with a Curie-type upturn at low temperatures is observed (Figure 7). The ferromagnetic behavior is indicated by the change of magnetic susceptibility χ(T) with field. The measured magnetization vs field *M*(H) at temperatures *T* = 2, 50, 150, and 300 K shows ferromagnetism with a narrow hysteresis and saturation of magnetization (Figure S6). The difference in susceptibility at high fields corresponds to an equivalent amount of 0.026 at. % Fe, which could only be explained by presence of ferromagnetic impurities. After subtracting the ferromagnetic impurity and the sample holder contributions from the overall signal, the remaining signal is paramagnetic. By fitting the remaining signal with the Curie law, the effective magnetic moment value μ_eff_ = 0.1 μ_B_ is obtained. This value is too low to describe a paramagnetic Cr ion in the bulk of the sample and can only be attributed to a small amount of unidentified paramagnetic impurities close to the detection limit of PXRD. Therefore, we believe that LiSr_2_[CrN_3_] is diamagnetic, which correlates well with both the expected absence of unpaired electrons in the non-magnetic ground state of chromium(IV), and the results of the crystal structure refinement.

## Conclusions

LiSr_2_[CrN_3_] is a new di-cationic nitridochromate(IV) containing slightly distorted [CrN_3_]^5–^ pseudo-trigonal pyramidal units. Magnetic measurements, vibrational spectroscopy, as well as shorter Cr–N distances in comparison with reported nitridochromates(III) indicate the presence of Cr(IV). Band structure calculations reveal that the compound is non-magnetic and a semiconductor with a band gap of 1.19 eV. The bonding situation is characterized by highly polar N-dominated N–Cr and Cr-dominated multi-atom bonds in line with the description as a nitridochromate.

## Supporting Information Available

Experimental and crystallographic details, calculation methods, Rietveld refinement, and expected internal vibrational modes for [CrN_3_]^5–^ anion (PDF)
