# Supplementary material for: Nitridochromate(IV): LiSr2[CrN3]
Source: Inorg Chem. 2023 Aug 3;62(32):12940–6. doi: 10.1021/acs.inorgchem.3c01697 (PMC10428211; doi:10.1021/acs.inorgchem.3c01697)
Supplement: Supplementary file 1 — ic3c01697_si_001.pdf [file ic3c01697_si_001.pdf]

Supporting information

A nitridochromate(IV): $LiSr_2[CrN_3]$

Natalia Gloriozova,[†] Yurii Prots,[†] Franziska Jach,[†,‡] Mitja Krnel,[†] Matej Bobnar,[†,§] Alim Ormeci,[†] Yuri Grin,[†] Peter Höhn[,†]*

[†]Max-Planck-Institut für Chemische Physik fester Stoffe, Nöthnitzer Straße 40, 01187 Dresden, Germany

[‡]Faculty of Chemistry and Food Chemistry, Technische Universität Dresden, 01062 Dresden, Germany

current address: Fraunhofer Institute for Integrated Systems and Device Technology IISB, Schottkystraße 10, 91058 Erlangen, Germany

[§]current address: Jožef Stefan Institute, Jamova cesta 39, 1000 Ljubljana, Slovenia

Corresponding author: Peter Höhn *peter.hoehn@cpfs.mpg.de*

Table of Contents

Experimental details S3

Calculation methods S5

Table S1. Synthesis of $LiSr_2[CrN_3]$ crystals S6

Table S2. Atomic coordinates and equivalent (isotropic) displacement parameters in $LiSr_2[CrN_3]$ according to single crystal refinement S7

Table S3. Anisotropic displacement parameters in $LiSr_2[CrN_3]$ according to single crystal refinement S8

Table S4. Interatomic distances in $LiSr_2[CrN_3]$ according to single crystal refinement S9

Figure S1. Rietveld refinement of $LiSr_2[CrN_3]$ powder sample S10

Figure S2. Crystal structure of $LiSr_2[CrN_3]$ (Cr – red, Sr – yellow, N – green, Li – blue). Anisotropic displacement parameters correspond to 90 % probability S11

Figure S3. Coordination of strontium and nitrogen atoms S11

Figure S4. The (*l*, *m*)-decomposed electronic DOS of $LiSr_2[CrN_3]$ computed with the quantization axis perpendicular to the plane of N-triangle S12

Table S5. Calculated and observed frequencies in Raman spectra of $LiSr_2[CrN_3]$ S13

Table S6. Expected internal vibrational modes for $[CrN_3]^{5-}$ anion within $LiSr_2[CrN_3]$ crystal structure according to molecular site group analysis S14

Figure S5. Infrared and Raman spectra of $LiSr_2[CrN_3]$....................S15

Figure S6. Field-dependent magnetization $M(H)$ of $LiSr_2[CrN_3]$ S16

References S17

Experimental details

All sample manipulations were performed in inert atmosphere using an argon-filled glove box ($\rho(O_2) / \rho o < 0.1$ ppm, $\rho(H_2O) / \rho o < 0.1$ ppm) due to the extreme sensitivity of both the starting materials and the final products to moisture and air.

The following starting materials were used: lithium rod (Alfa, 99.9 %), chromium powder (Alfa, 99.97 %), sodium azide powder (Sigma, 99.5%), strontium nitride (Sr_2N) powder, lithium nitride (Li_3N) powder.

Strontium nitride (Sr_2N) was synthesized by annealing strontium metal (Alfa, dendritic pieces, 99.8 %) under nitrogen flow (Praxair, 99.9999 %, additionally purified by molecular sieves and BTS-catalyst) at 973 K for 12 h, followed by cooling down to 573 K.

Lithium nitride (Li_3N) was prepared from lithium rod (Alfa, 99.9 %) under nitrogen flow (Praxair, 99.9999 %, additionally purified by molecular sieves and BTS-catalyst) at 573 K for 10 h, followed by cooling down to room temperature.

Microcrystalline $LiSr_2[CrN_3]$ was synthesized from a mixture of pelletized Sr_2N, CrN, and Li_3N with molar ratio 0.7:1:2.6 in a sealed tantalum ampule at 1023 K for 36 h (heating / cooling rate 100 K/h and 20 K/h, respectively). Several grinding, repelletizing and heating cycles (at 1023 K for 120 h each heating cycle) with additional Sr_2N and Li_3N were needed to obtain nearly a single-phase sample with some unspecified impurities and to remove Cr impurities.

Crystals of $LiSr_2[CrN_3]$ were synthesized from Sr_2N, Cr, Li_3N, and Li in molar ratio 1:1.8:2.8:17 using Li_3N as nitrogen source and Li acting as flux. The sample mixture of roughly 0.7 g was placed in sealed tantalum ampule with sieve inside and was heated to 1023 K for 2 h (heating rate 100 K/h). After cooling down to 573 K with rate 1 K/h the sample was centrifuged with 3000 min^{-1} to allow proper separation of crystals from the flux. To check the phase width and for other phases in the system, several more experiments were performed using the same technique, but employing different starting materials, molar ratios or thermal regimes (for details see Table S1).

Crystal structure and composition of the title compound were refined from single-crystal X-ray diffraction data recorded at room temperature on a Rigaku AFC7 diffractometer equipped with a Saturn 724+ CCD area detector (Mo Kα radiation). Suitable single crystals were selected in a glove box and enclosed in wire-sealed glass capillaries. Crystal structure solution was accomplished by

using the programs SHELXS-2019[1] and SHELXL-2019[2] (Table S2, S3, S4). Refinement in $P\,2_1/m$ did not succeed, but resulted in large anistropic displacement parameters for all atomic positions and the one atom (N3) not located on the pseudo-mirror plane is refined with half occupancy.

Further details of the crystal structure investigations may be obtained from FIZ Karlsruhe, 76344 Eggenstein-Leopoldshafen, Germany (fax: (+49)7247-808-666; e-mail: crysdata@fiz-karlsruhe.de, on quoting the deposition numbers CSD-2264610).

Graphical representations of the structure were created in Diamond.[3]

Powder X-ray diffraction patterns of finely ground powders were collected on a Huber G670 imaging plate Guinier camera using a curved germanium (111) monochromator and Cu-$K\alpha_1$ radiation in the range $4° \leq 2\theta \leq 100°$ with an increment of 0.005° at room temperature. Powder samples were enclosed between two Kapton foils to reduce contact with air. Preliminary data processing was performed within the WinXPow suite of programs.[4] The software package Jana2006[5] was used for Rietveld refinement of the structure.

Raman spectra of crystals of $LiSr_2[CrN_3]$ were collected at room temperature in the range 4000 – 50 cm^{-1} using a HR Evolution spectrometer equipped with a Laser Quantum Ventus diode laser (532 mm) and a Synapse CCD detector. The spectral resolution was about 0.42 cm^{-1}. Suitable crystals were sealed in quartz capillaries for protection against air and moisture inside.

Infrared (IR) spectra of finely ground crystals and powder samples were recorded in Attenuated Total Reflectance (ATR) mode using a PerkinElmer UATR-Two FTIR spectrometer inside the glove box in the range 4000 – 450 cm^{-1}. For each measurement background was corrected by the measurement with the empty setup. No signals indicating moisture have been detected by IR or Raman spectroscopy in any of the samples.

Magnetic measurements were performed on finely ground powder of the title compound with a mass equal to 135.2 mg enclosed in a sealed pre-calibrated quartz tube under 400 mbar of He using a Quantum Design MPMS XL-7 SQUID magnetometer equipped with a 7 T magnet within the temperature range 1.8 – 300 K. All data were corrected for the diamagnetic contribution of the quartz tube.

Calculation methods

Electronic structure and chemical bonding analysis were investigated by using the all-electron full-potential local orbital (FPLO) method.[6] The Fritz-Haber Institute ab initio molecular simulations (FHI-aims) method[7] was employed to calculate the phonon density of states (DOS) and to analyze the characters of the modes at the Γ point. Exchange-correlation effects were taken into account by either the local density approximation (LDA) or the generalized gradient approximation (GGA) to the density functional theory, the former as parametrized by Perdew and Wang,[8] the latter by Perdew, Burke and Ernzerhof (PBE).[9] The Brillouin zone was sampled by a mesh of 14 x 14 x 10 (or equivalent) in this study. Experimentally determined crystal structure data and LDA were used for the electronic structure and chemical bonding calculations. The chemical bonding situation in position space was investigated within the framework of combined topological analysis of electron density (ED) and electron localizability indicator (ELI)[10]. This approach is based on the quantum theory of atoms in molecules (QTAIM).[11] ELI was calculated in the ELI-D representation[12] by a module implemented in the FPLO package.[13] The atoms participating in a bond were determined by applying the basin intersection technique.[14] The topological analysis was carried out by the program Dgrid.[15] The fully-optimized (using the GGA) crystal structure was adopted for the lattice dynamics calculations. The force constants were computed by the finite difference/frozen phonon method employing a $2 \times 2 \times 2$ supercell and an atomic displacement of 0.001 Å.

Table S1. Synthesis of $LiSr_2[CrN_3]$ crystals.

sample	starting materials / molar ratio					annealing temperature / K	cooling rate / K/h	products
	Sr_2N	Cr	Li_3N	NaN_3	Li			
1	1	1.8	2.8	–	17	1023	1	$LiSr_2[CrN_3]$
2	1	3.8	6	–	35	1023	1	$LiSr_2[CrN_3]$, $Sr_3[CrN_3]H$
3	1	1.8	–	0.3	17	1023	0.5	$LiSr_2[CrN_3]$, $Sr_3[CrN_3]$

Table S2. Atomic coordinates and equivalent (isotropic) displacement parameters in $LiSr_2[CrN_3]$ according to single crystal refinement.

atom	Wyckoff site	*x/a*	*y/b*	*z/c*	$U_{eq/iso}$ (Å^2)
Sr1	2*a*	0.27808(11)	0.80156(15)	0.98791(9)	0.0098(2)
Sr2	2*a*	0.16371(12)	0.32178(14)	0.65549(9)	0.0113(2)
Cr1	2*a*	0.7832(2)	0.7730(3)	0.76586(15)	0.0073(3)
N1	2*a*	0.7724(11)	0.8049(18)	0.9920(8)	0.0106(11)
N2	2*a*	0.0503(11)	0.8407(17)	0.6684(8)	0.0112(14)
N3	2*a*	0.5850(14)	0.5714(17)	0.6611(10)	0.0168(15)
Li1	2*a*	0.362(3)	0.828(4)	0.537(2)	0.019(3)

Table S3. Anisotropic displacement parameters in $LiSr_2[CrN_3]$ according to single crystal refinement.

atom	U_{11}	U_{22}	U_{33}	U_{23}	U_{13}	U_{12}
Sr1	0.0095(3)	0.0051(4)	0.0149(3)	0.0002(3)	0.0011(2)	–0.0003(3)
Sr2	0.0154(3)	0.0083(4)	0.0104(3)	0.0000(3)	0.0015(2)	–0.0015(3)
Cr1	0.0080(5)	0.0054(7)	0.0086(5)	0.0000(4)	0.0022(3)	–0.0002(5)
N1	0.016(3)	0.004(3)	0.012(3)	–0.002(3)	0.004(2)	0.000(3)
N2	0.009(3)	0.010(4)	0.014(3)	0.002(3)	0.004(2)	–0.003(2)
N3	0.016(3)	0.016(4)	0.018(4)	–0.003(3)	–0.001(3)	–0.004(3)
Li1	0.021(7)	0.016(9)	0.018(6)	–0.006(7)	–0.002(5)	0.005(8)

Table S4. Selected interatomic distances in $LiSr_2[CrN_3]$ according to single crystal refinement.

atoms		*d*, Å
Cr1	–N1	1×1.716(6)
	–N2	1×1.724(6)
	–N3	1×1.717(8)
Sr1	–N1	1×2.693(9)
	–N1	1×2.729(9)
	–N1	1×2.752(6)
	–N1	1×2.817(6)
	–N2	1×2.685(6)
	–N3	1×3.087(8)
Sr2	–N1	1×2.669(6)
	–N2	1×2.668(9)
	–N2	1×2.676(6)
	–N2	1×2.866(9)
	–N3	1×2.702(8)
Li1	–N2	1×2.032(16)
	–N3	1×2.02(2)
	–N3	1×2.05(2)
Sr1	–Cr1	3.1704(16)
	–Cr1	3.1705(13)
	–Cr1	1×3.3335(13)
	–Cr1	1×3.4216(16)
Sr2	–Cr1	1× 3.2125(13)
	–Cr1	1× 3.3499(15)
Sr1	–Li1	1×3.449(15)
Sr2	–Li1	1× 3.03(2)
	–Li1	1× 3.065(15)
	–Li1	1× 3.08(2)
Cr1	–Li1	1×2.868(15)
Li1	–Li1	2×3.161(16)

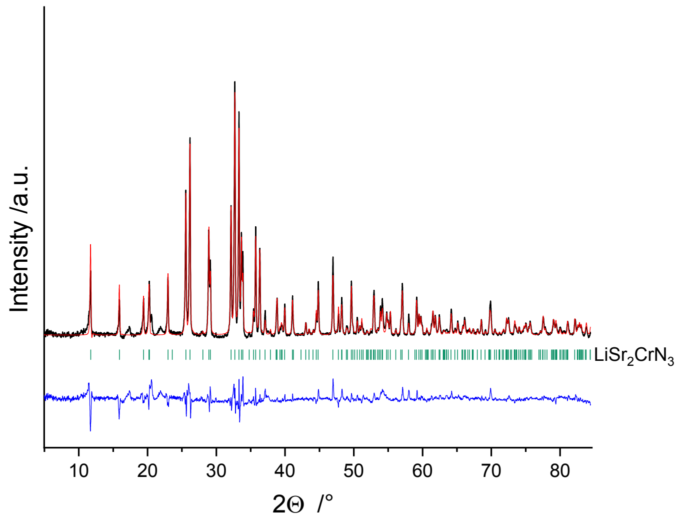

Figure S1. Rietveld refinement of $LiSr_2[CrN_3]$ powder sample with experimental points shown in black, calculated intensity after Rietveld refinement in red, and difference curve in blue (Cu Kα_1).

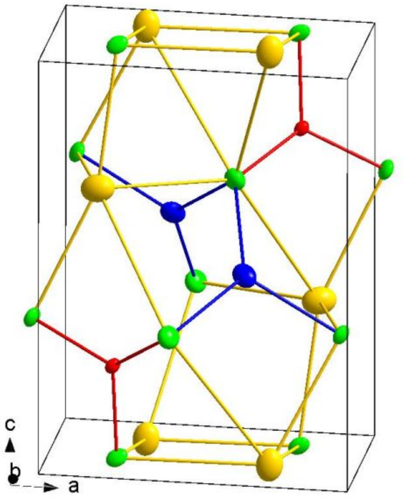

Figure S2. Crystal structure of $LiSr_2[CrN_3]$ (Cr – red, Sr – yellow, N – green, Li – blue). Anisotropic displacement parameters correspond to 90 % probability.

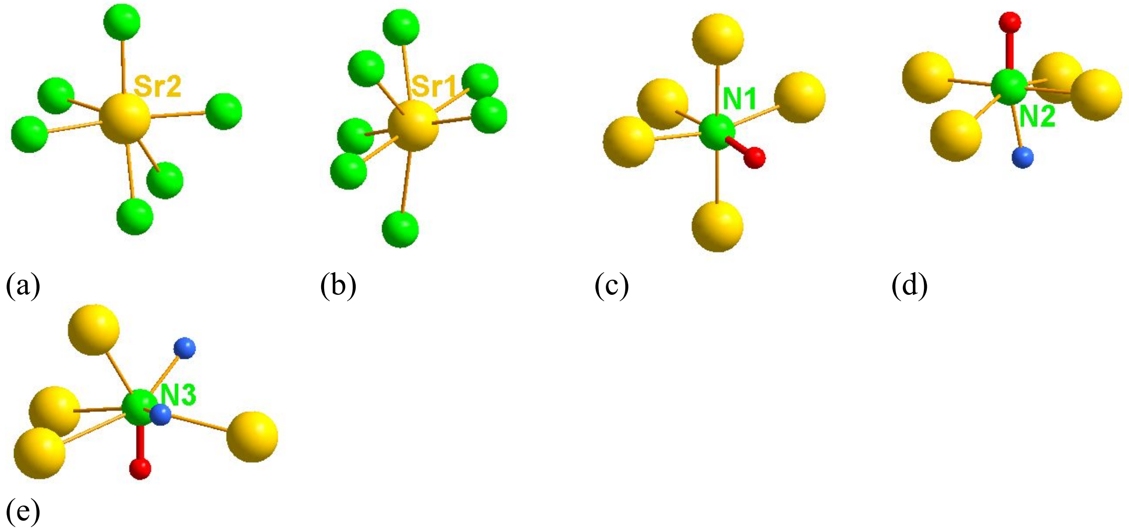

Figure S3. Coordination of (a, b) strontium and (c, d, e) nitrogen atoms (Cr – red, Sr – yellow, N – green, Li – blue).

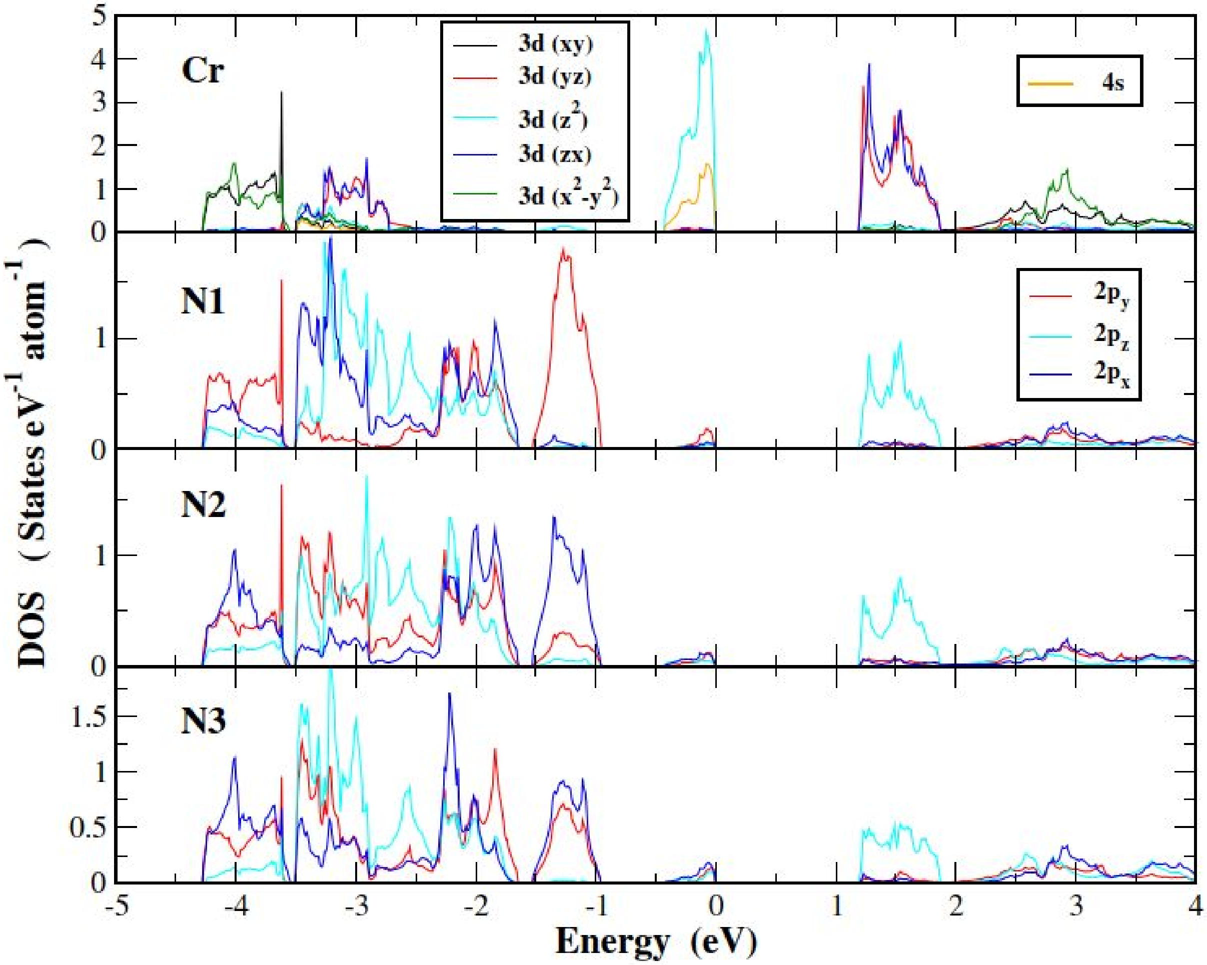

Figure S4. The (*l*, *m*)-decomposed electronic DOS of $LiSr_2[CrN_3]$ computed with the quantization axis perpendicular to the plane of N-triangle. For Cr, five 3d as well as the 4s orbital, and for the N atoms three 2p orbital contributions are plotted. For clarity, orbitals 3d ($3z^2 - r^2$) are marked as 3d (z^2).

Table S5. Calculated and observed frequencies in Raman spectra of $LiSr_2[CrN_3]$.

calculated frequencies /cm^{-1}	observed frequencies /cm^{-1}
852	877
845	858
844	844
837	–
831	–
829	822
485	501
477	483
474	–
466	–
407	–
400	402
396	–
385	–
337	344
294	298
280	274
258	–
251	–
244	245

Table S6. Expected internal vibrational modes for $[CrN_3]^{5-}$ anion within $LiSr_2[CrN_3]$ crystal structure according to molecular site group analysis.

Molecular vibration	Molecular point group	Molecular selection rules	Site symmetry	Factor group	Solid state selection rules	Number of expected bands	
	C_{3v}		1	C_2		IR	Raman
$\upsilon_s(CrN_3)$	A_1	IR, R	*A*	*A*	IR, R		
				B	IR, R		
$\upsilon_{as}(CrN_3)$	*E*	IR, R	*A*	*A*	IR, R	6	6
				B	IR, R		
			A	*A*	IR, R		
				B	IR, R		
$\delta_s(NCrN)$	A_1	IR, R	*A*	*A*	IR, R		
				B	IR, R		
$\delta_{as}(NCrN)$	*E*	IR, R	*A*	*A*	IR, R	6	6
				B	IR, R		
			A	*A*	IR, R		
				B	IR, R		

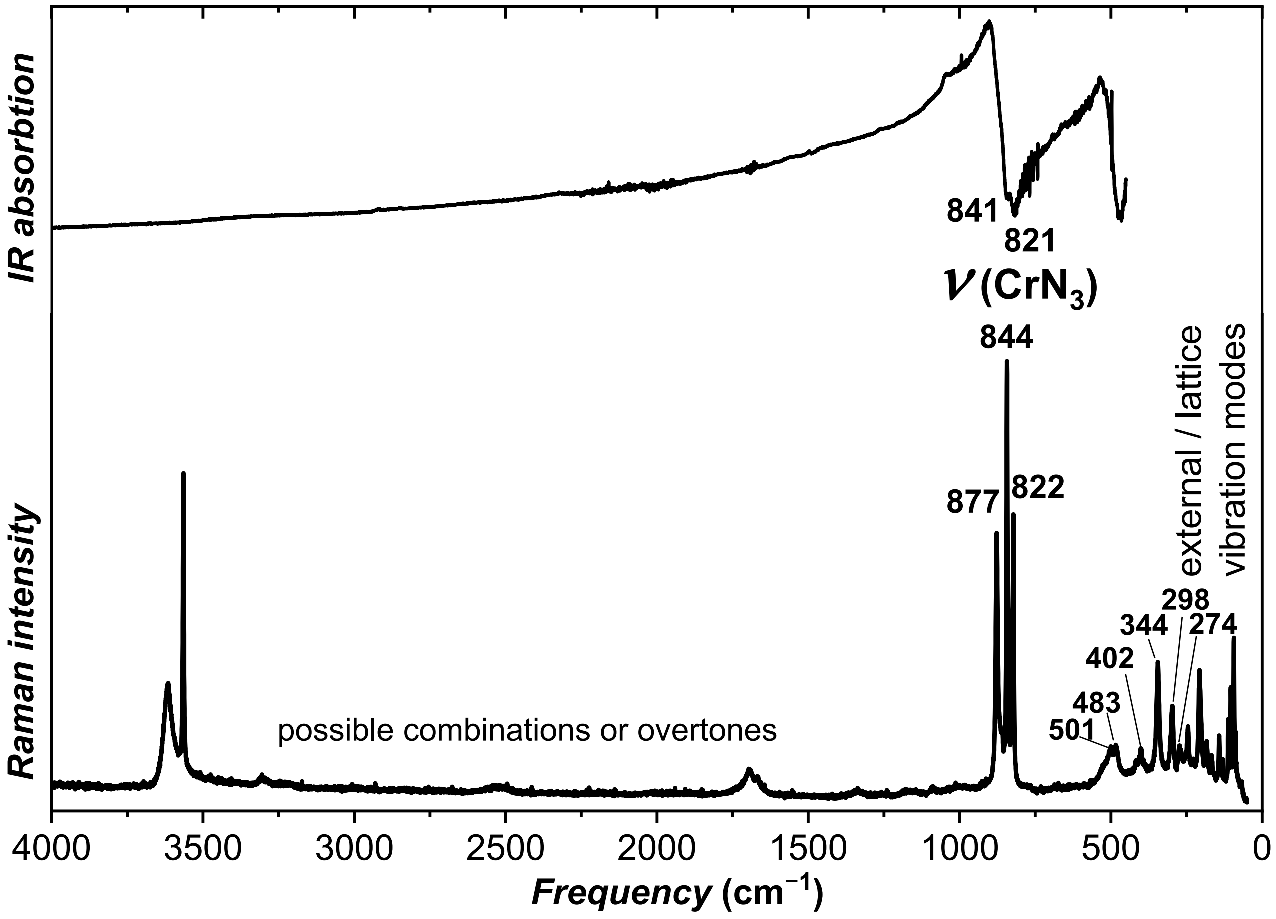

Figure S5. Infrared (top) and Raman spectra (bottom) of $LiSr_2[CrN_3]$.

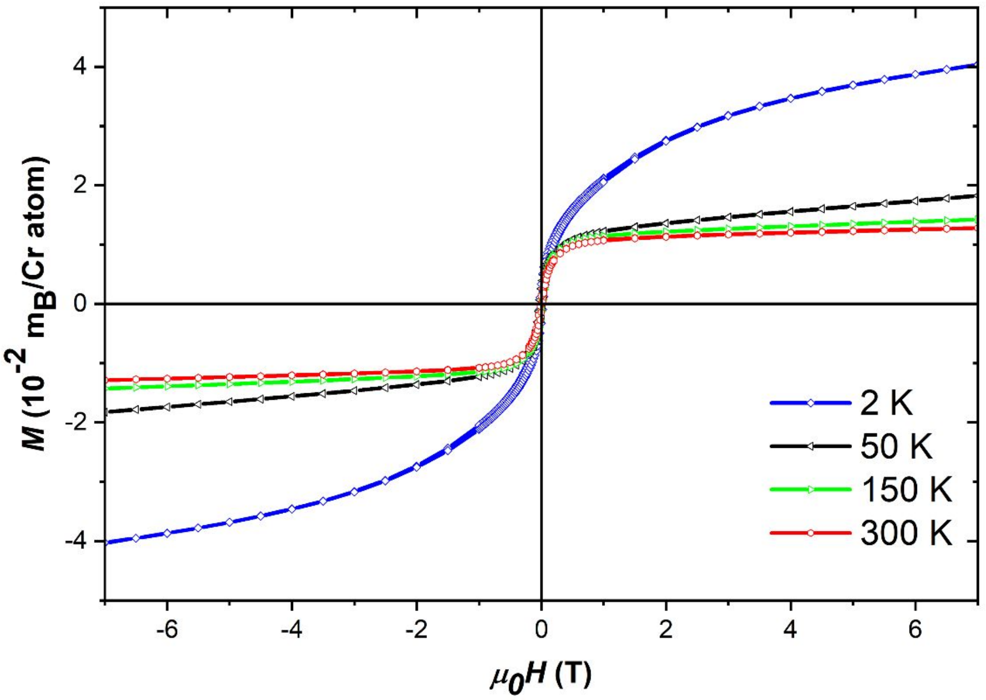

Figure S6. Field-dependent magnetization $M(H)$ measured at T = 2 K, 50 K, 150 K and 300 K.

References

1. Sheldrick, G., SHELXS-2019, program for crystal structure solution. University of Göttingen, *Göttingen, Germany* **2019**.
2. Sheldrick, G. SHELXL-2019, Program for the Refinement of Crystal Structures; University of Göttingen, *Göttingen, Germany* **2019**.
3. Impact, C. Diamond-crystal and molecular structure visualization. Kreuzherrenstr. 102, 53227 *Bonn, Germany*, **2017**.
4. WinXPOW, D. S. version 2.21. *Stoe & Cie GmbH, Darmstadt, Germany* **2007**.
5. Petříček, V.; Dušek, M.; Palatinus, L. Crystallographic computing system JANA2006: general features. *Z. Kristallogr.-Cryst. Mater.* **2014,** 229 (5), 345-352.
6. Koepernik, K.; Eschrig, H. Full-potential nonorthogonal local-orbital minimum-basis band-structure scheme. *Phys. Review B* **1999,** 59 (3), 1743.
7. Blum, V.; Gehrke, R.; Hanke, F.; Havu, P.; Havu, V.; Ren, X.; Reuter, K.; Scheffler, M. Ab initio molecular simulations with numeric atom-centered orbitals. *Comput. Phys.Commun.* **2009,** 180 (11), 2175-2196.
8. Perdew, J. P.; Wang, Y. Accurate and simple analytic representation of the electron-gas correlation energy. *Phys. Rev. B* **1992,** 45 (23), 13244.
9. Perdew, J. P.; Burke, K.; Ernzerhof, M. Generalized gradient approximation made simple. *Phys. Rev. Lett.* **1996,** 77 (18), 3865.
10. Grin, Yu.; Savin, A.; Silvi, B., In The Chemical Bond: Fundamental Aspects of Chemical Bonding, edited by G. Frenking & S. Shaik, Chapter 10. In Weinheim: Wiley-VCH: 2014.
11. Bader, R. F. Atoms in molecules. *Acc.Chem. Res.* **1985,** 18 (1), 9-15.
12. Kohout, M. A measure of electron localizability. *Int. J. Quantum Chem.* **2004,** 97 (1), 651-658.
13. Kohout, M. Bonding indicators from electron pair density functionals. *Faraday Discus.* **2007,** 135, 43-54.
14. Wagner, F. R.; Bezugly, V.; Kohout, M.; Grin, Yu. Charge decomposition analysis of the electron localizability indicator: a bridge between the orbital and direct space representation of the chemical bond. *Chem. – Eur. J.* **2007,** 13 (20), 5724-5741.
15. Ormeci, A.; Rosner, H.; Wagner, F.; Kohout, M.; Grin, Yu. Electron localization function in full-potential representation for crystalline materials. J. *Phys. Chem. A.* **2006,** 110 (3), 1100-1105.
16. Raub, S.; Jansen, G. A quantitative measure of bond polarity from the electron localization function and the theory of atoms in molecules. *Theor. Chem. Acc.* **2001,** 106 (3), 223-232.
17. Kohout, M. DGrid 4.6., *Dresden, Germany* **2011**.
